# Narratives of Adults Registered Female at Birth Who Started a Medical Transition and Later Detransitioned

**DOI:** 10.1007/s10508-025-03083-9

**Published:** 2025-04-07

**Authors:** Jane Lomax, Catherine Butler

**Affiliations:** Department of Psychology, University of Bath, Bath, BA2 7AY UK

**Keywords:** Gender dysphoria, Transgender, Gender affirmative care, Detransition, Narrative analysis

## Abstract

The visibility and presence of people who have detransitioned following a gender transition is growing, with an increase in research on the needs and experiences of this group. This study presents a thematic narrative analysis of interviews from six females (M = 25.5 yrs; range = 21–32 yrs). All detransitioned after having at least one gender-affirming medical or surgical treatment as part of a gender transition in the UK. Four narrative themes were developed to capture how they made sense of their detransition: (1) the limits of medical transition, (2) the longer-term health implications, (3) the social limits of transition, and (4) detransition as an ongoing process. Participants discussed a range of emotional, practical, and other support needs, largely unmet by healthcare or other services. These findings highlight the importance of ensuring that people have realistic expectations of transition as part of a holistic assessment process. Long-term health outcome research is also needed, addressing the impact of testosterone on female anatomy and health specifically. Further clinical implications with those considering transition or detransition are discussed.

## Introduction

Transgender and gender diverse (TGD) people experience a mismatch between their gender identity and their registered birth sex (Coleman et al., 2022). Some also experience gender dysphoria (GD), distress associated with this mismatch (Zucker et al., 2016), which may be social and/or body related (Vandenbussche, 2022). To alleviate this, many seek a gender transition, making social (e.g., changing name or pronouns, altering appearance), legal (e.g., legal name or sex marker change), and/or medical changes. Medical transitions include gender-affirming medical or surgical treatments (GAMSTs), seeking to align the appearance of the body with the experienced sense of self through hormones or surgeries (Hall et al., 2021; Keo-Meier & Ehrensaft, 2018).

There is not a consistent definition of detransition across researchers (Exposito-Campos et al., 2023; Jorgensen, 2023), but most include the process of discontinuing or reversing a medical transition, often in connection with a change in how the individual identifies or conceptualizes their sex or gender (e.g., MacKinnon et al., 2023b). This may be complete, partial, definitive, or temporary (Exposito-Campos et al., 2023). Therefore, there can also be medical, legal, and social components to detransition, with varying combinations creating unique detransition experiences for individuals (Hildbrand-Chupp, 2020). There is also heterogeneity in definition among those who stop or reverse medical treatment, with some people considering themselves as “detrans,” some re-identifying with their sex assigned at birth and others continue with a TGD identity (Expósito-Campos, 2021; MacKinnon et al., 2023b). Hildbrand-Chupp (2020) suggests breaking detransition down into three “types”: detransition as an act (to stop or reverse some aspect of transition but maintain a transition identity), detransition as identity (related to a change in gender understanding and a re-identification with natal sex), and detransition following a negative transition experience (and linked to regret). It should be noted that for prepubescent youth who express gender diversity related changes (e.g., social transition), but do not receive medical interventions, a cessation of such requested changes is referred to as “desistance” (Cohen et al., 2022; Exposito-Campus et al., 2023; Jorgensen, 2023). However, desistance is a clinical term and not used as a term of self-identification (Hildebrand-Chupp, 2020).

Data from previous cohorts suggest low detransition rates (e.g., 2.2.%, Dhejne et al., 2014; 0.3–0.6%, Wiepjes et al., 2018). However, as discussed, varied definitions of detransition are used in the literature (Exposito-Campos, et al., 2023) and many of these studies are based on adults who “completed” medical transition including genital reconstructive surgery (White Hughto & Reisner, 2016), with less known about those who do not access all available GAMSTs (Hall et al., 2021). Researchers have also found high rates of patients lost to follow up, over 40% in some studies (Exposito-Campos et al., 2023). While not all those who are lost to follow up will have detransitioned, detransitioners may be more likely not to return to clinics (e.g., due to feelings of shame, stigma and distrust of clinicians) and therefore their outcomes will not be recorded (MacKinnon et al., 2022a). In addition, most studies do not follow up beyond 5 years (MacKinnon et al., 2023a), most only 1–2 years (Exposito-Compas et al., 2023), yet recent research has found that people are more likely to detransition 5–10 years after transitioning (Gribble et al., 2023; MacKinnon et al., 2023a, 2023b); therefore, an accurate picture is not being collected.

The demographics of people presenting to gender care services has changed significantly in recent years, shifting from predominantly adult males to majority adolescent females (Butler & Hutchinson, 2020; Jorgensen, 2023). Thompson et al.’s (2022) systematic review found that around 64% of GD diagnoses are currently made in patients assigned female at birth. The number of young people has risen sharply (de Graaf et al., 2018; GIDS, 2022); an analysis of private and public health insurance data between 2017 and 2021 found that GD diagnoses nearly tripled in the USA, with over 14,000 young people aged 17 and younger starting to take gender-affirming hormones (Respaut & Terhune, 2022). Finally, gender diversity is increasingly associated with complexity such as autism, trauma, and mental health issues (e.g., Kaltiala-Heino et al., 2015; Paz-Otero et al., 2021). The drivers for these changes are not fully understood (Butler et al., 2022), making outcomes and detransition rates for those seeking help in recent years difficult to predict (Hutchinson et al., 2020; Jorgensen, 2023; MacKinnon et al., 2023a). Data from more recent cohorts suggest much higher rates of detransition than those previously found (between 7 and 30%) (Hall et al., 2021; Roberts et al., 2022); however, these studies also had inconsistent definitions of detransitioning, therefore, reliable estimates remain elusive.

As well as cohort changes, there have been changes in the medical approach to gender transition. Prior to September 2022, those wishing to transition had to complete a “real life test,” requiring them to live for a period of time (sometimes up to two years) in their felt gender role, before they are given access to any medical interventions. This idea was based on a binary view of gender, where transition was viewed as changing to the “opposite” sex (Katz-Wise et al., 2023) reflecting transnormative views with a limited understanding of nonbinary identities or gender fluidity (MacKinnon et al., 2023b). However, in September 2022, the 8th version of the World Professional Association of Transgender Health’s (WPATH) Standards of Care was published, which removed the real life test as a requirement. Transgender medical care shifted away from a gender binary model, to supporting people to fulfill their individualized gender identity or “gender embodiment goals” (Coleman et al., 2022). This fits with the lived experience of TGD people. For example, Katz-Wise et al. (2023) found that over time, it was equally common for transgender youth to move to a nonbinary gender identity, as to a binary one. Cohen et al. (2022) found that there were more shifts in gender-related medical requests among nonbinary participants, and almost half of their participants changed their gender identity during their study. The participants in MacKinnon et al. (2023b) reported multiple gender identity shifts alongside detransitioning. In addition to shifts in identity, research has also found a shift in embodiment goals after medical gender interventions began (MacKinnon et al., 2023b). It should be noted that early research on detransition (e.g., Kuiper & Cohen-Kettenis, 1998; Pfäfflin & Junge, 1998) took place before these cohort, medical, and definitional changes, and so may not be relevant today. In addition, this early research had a methodological focus on understanding detransition to reduce its occurrence, as another means of gatekeeping transition, whereas current research views those who detransition as a subgroup of people who have unique lived experiences and medical/mental health care needs (Hildbrand-Chupp, 2020).

Detransition has been controversial, with concerns it may be used to undermine needed healthcare for TGD people, or that it fuels anti-transgender rhetoric (MacKinnon et al., 2021; Slothouber, 2020). Detransitioning is stigmatized, and people have reported a lack of support and rejection from others because of their detransition (Vandesbussche, 2022), as well as a lack of professional care from mainstream and LGBTQ + organizations (MacKinnon et al., 2023b). This has resulted in some people who have detransitioned avoiding health care because of feelings of shame or stigma (Littman, 2021; MacKinnon et al., 2023c; Vandesbussche, 2022), or a lack of trust and feeling let down because of prior encouragement to transition, without careful assessment and exploration of alternatives for treating GD (Exposito-Campus, 2021; Gribble et al., 2023; Sanders et al., 2023).

Detransition poses significant professional and bioethical challenges for the medical care of people with GD (Expósito-Campos, 2021) and the visibility of detransitioned people has increased, particularly online (Littman, 2021; MacKinnon et al., 2023b; Marchiano, 2020). While the current WPATH Standards of Care (Coleman et al., 2022) acknowledge the need to support people considering detransition, they have been criticized for failing to provide substantive guidelines (GENSPECT, 2022). Meanwhile, detransitioners consistently report requiring more support and information about their mental health and medical needs (e.g., stopping hormones, surgical reversal, reproductive capacity, etc.; Gribble et al., 2023; MacKinnon et al., 2022b; Sanders et al., 2023; Vandenbussche, 2022). When this is found lacking because of a lack of knowledge or services, they may choose to disengage with services (MacKinnon et al., 2023a, 2023b), turning to other detransitioners via social media instead (Littman, 2021; MacKinnon et al., 2023b).

The detransition steps taken vary, they may include reversing some, or all, the medical, legal, or social changes where this is possible (Vandesbussche, 2022). A myriad of factors have been identified that might contribute to a person’s decision to detransition, which Exposito-Campos et al. (2023) have grouped into five categories: psychological, medical, social, cultural, and ideological. Psychological factors included doubts/fluctuations in gender identity, no improvement or worsening of mental health, resolution or alternative ways of coping with gender dysphoria. Sanders et al. (2023) also found some people felt inauthentic in their transitioned gender. Whereas Littman et al. (2024) found some people become more comfortable identifying with their natal sex, Cohen et al. (2022) found that some people stopped medical interventions when their gender-related goals were met. Medical factors included physical health concerns or issues, fertility concerns and dissatisfaction with the results of medical treatment. Social reasons were more external, including a lack of support and understanding from others, lack of financial resources, legal reasons and difficulty accessing medical treatment. Cultural reasons included complying with ideas from one’s own culture, or discovering an intersection between gender dysphoria and internalized misogyny or homophobia. Finally, ideological reasons included rejecting gender stereotypes, a change in one’s ideological beliefs, or realizing the impossibility of changing one’s birth sex.

MacKinnon et al. (2023b) interviewed 28 participants with experiences of detransition, and grouped their reasons for detransition (all reflected in Exposito-Campos et al.’s (2023) categories) into four “pathways to detransition:”

- 1. Discrimination and TGD identity repression: these external reasons were experienced just by trans women, who later went on to retransition or identify as nonbinary.
- 2. Gender-affirming hormone discontinuation and evolving identity: these participants might have experienced medical side effects, but viewed their detransition as an opportunity for growth and re-evaluation of embodiment goals.
- 3. Binary transition to nonbinary detransition: participants considered they initially pursued a binary gender transition because of transnormative cultural narratives and pressure to medically transition. Some of these participants mentioned feelings of regret.
- 4. Detrans identity development within the social context: a change in social context created an opportunity to detransition and a chance to overcome feelings of shame associated with this, this including exposure to new online detrans communities, particularly for natal females.

These pathway descriptors are useful to illustrate the complexity of detransition, that it involves multiple intersecting internal and external influences and a variety of emotional responses.

Continuing support is needed after detransition, as while some people can feel positive (Pullen Sansfaçon et al., 2023) and view transition/detransition as an important developmental step (Littman, 2021; Pullen Sansfaçon et al., 2023; Turban & Keuroghlian, 2018), others report ambiguous feelings or distress (Pullen Sansfaçon et al., 2023), sometimes because of physical changes made during a transition (MacKinnon et al., 2022a; Sanders et al., 2023). For some, detransitioning can bring the return of gender dysphoria (MacKinnon, et al., 2023a). This gender dysphoria associated with their birth sex may return, or it may be iatrogenic, following changes to the body from medical intervention (MacKinnon et al., 2023a), or distress at still being perceived as transgender following detransition because of physical changes (referred to as “reverse dysphoria;” MacKinnon et al., 2022a). Some people regret aspects of their transition (e.g., surgical results), and may then detransition, while others might not. Some people detransition without experiencing regret (Jorgensen, 2023; MacKinnon et al., 2022b), and for others regret and satisfaction have been found to coexist (Pullen Sansfacon et al., 2023). Expósito-Campos et al. (2023) stressed that in the older literature, detransition was often described as “regret,” regardless of whether the person actually expressed the emotion of regret or said that this informed their decision. In addition, these authors point out that feelings of regret will vary in intensity, duration, and in what triggers it (e.g., post-intervention medical complications or an identity change). It should therefore not be assumed that detransitioners will experience regret at having transitioned.

It is therefore clear that detransitioning is an extremely complex phenomenon, with shifting definitions, changing sociocultural landscapes, and new models of care. There has been an increase in research in this area in recent years, and increasing calls for more qualitative research to further understanding of the diverse experiences of detransitioners, with a view to ultimately improving healthcare (Hall et al., 2021; Hildbrand-Chupp, 2020; Littman, 2021; MacKinnon et al., 2023a). Researching the experiences of detransitioners fits with the priorities of trans health experts (Veale et al., 2022). The current study answers this call, aiming to gain a nuanced understanding of the personal narratives and meaning making of people who have detransitioned in the UK. This study focused on birth-registered females, due to their increased presentation to services.

## Method

### Participants

Six participants were interviewed, living across the UK, aged 21–32 years (M = 25.5 years) at the time of interview. Preinterview, four participants identified themselves as female and two “preferred not to say.” At the end of the interview, participants were asked how they would like to be referred to in this report, and their pronoun and pseudonym preferences. All opted to be referred to as female (she/her pronouns), stating this was for clarity related to their view that they were biologically female, even if they still felt confused about gender identity at times, were sometimes gendered as male, or preferred not to think about gender in their daily lives. Four identified as White British, two as White (other). Three described their sexuality as lesbian, one as asexual/lesbian, and two as bisexual. Four had been diagnosed with autism or ADHD and all had experienced struggles with their mental health and/or GD. Two described gender trajectories beginning in childhood, two in adolescence, and two in adulthood, for GD and/or TGD identity. All had taken testosterone for between one and five years, starting between ages 17–24 years, and four had double mastectomies (ages unclear). They had detransitioned for between two months and five years.

### Procedure

To enhance understanding and knowledge construction, an expert by experience (registered female at birth who had stopped a medical transition, identified as female, and used either male or female pronouns) was recruited via Reddit and reviewed the research design and research materials.

Participants were recruited online via Twitter, the r/detrans subreddit, a Facebook gender discourse discussion group, and The UK Detransition Advocacy Network website. Inclusion criteria were being over 18, registered female at birth, fluent English, and having transitioned and detransitioned in the UK. Transition was defined as having started at least one GAMST, to increase the specificity of the sample. Detransition was defined as stopping a transition or self-defining as having detransitioned for any reason. Aiming for diversity of detransition narratives and identity inclusivity, the advert explicitly welcomed participants with any gender identity, gender expression, or sexual orientation (or none).

### Measures

After gaining written and verbal informed consent, the first author conducted individual, in-depth, virtual interviews on Microsoft Teams, using a life history approach (Wengraf, 2001). Participants were invited to tell their story of transition and detransition, all experiences that were important for them. A strength of this approach is that it allows participants to shape the story told, prioritizing their perspectives and including what they deem important. This compares to semi-structured interviews, where the researcher determines areas of interest (Mishler, 1986). The interviewer aimed to listen without interruption, providing facilitative support (e.g., attentive listening, nonverbal cues). Once the participant had finished their account, the interviewer asked follow-up questions about topics raised by the narrator in the language and order they had been raised, to elicit further, more detailed narratives (e.g., Can you tell me more about X? What happened after that?). If topics of interest to the researcher were not raised, follow-up questions were used to explore whether they were felt to be relevant to the story (e.g., relationship to the body, hopes/fears, sexuality, stigma, or discrimination). Reasonable adjustments were made to accommodate neurodiversity or mental health related needs (e.g., breaks, or increased facilitative questions to enable storytelling). Following the interview, all participants engaged in a debrief session to reflect on the relational and process elements of telling their story, to allow them to ask any questions, to explore their own theories and interpretations more explicitly, and to explore their hopes for sharing their story. This included reflection on the ethical challenges posed by the inherent power imbalance within the research process. Interviews were conducted between January and March 2021 and were 60–190 min long; participants were paid £15. Interviews were transcribed verbatim.

### Data Analysis

Thematic narrative analysis (TNA) was used to examine the way that people made sense of experiences and communicated about themselves and their evolving identities over time (Riessman, 2008). TNA offers a multilayered analysis, allowing for in-depth exploration of how individual experiences are negotiated in relation to wider sociocultural narratives, revealing the temporal, emotional, and contextual quality of lives (Braun & Clarke, 2013). To enhance understanding and knowledge construction, TNA seeks to identify themes regarding the way stories are told or constructed (Riessman, 2008). The following stages were used iteratively to facilitate date engagement: repeated listening, reading and re-reading the data; identifying narratives; analyzing the content of narratives (*what* is said); analyzing the structure (*how* things are said); creating narrative outlines; and developing narrative themes within and across accounts. The narrative themes were refined through discussion with the research team, including an expert by experience, acting as “critical friends” (Campbell et al., 2004).

The analysis aimed to stay close to the meanings and perspectives of participants, using their own words wherever possible (Riessman, 2008). Trustworthiness relates to how persuasive and plausible the analysis is, how well it is supported by evidence, whether any data contradicts the interpretation given and whether alternative interpretations have been considered (Riessman, 2008). However, narratives are also social practices that people perform and do in relation to others as opposed to something they have (Smith & Sparkes, 2006). Narratives are therefore cocreated and as cisgendered women, in practicing reflexivity, the authors met regularly and critically evaluated the influence of their life experiences, values, beliefs, and embodied selves on the research process (Couture et al., 2012; Sparkes & Smith, 2015). We questioned each other about assumptions when doing the analysis, the language we were using and assumptions behind this. We took care to allow time for these discussions and did further reading, which led to a deeper understanding of issues that we had not lived through ourselves. The individual narrative outlines and developing themes were shared with participants (although no responses were received), reviewed and discussed with the expert by experience and a clinician with experience of working with TGD young people. The expert by experience commented, based on their own experience and involvement in detransition forums, that the results resonated with their own and others’ experiences, and suggested areas in the analysis that they felt needed further expansion. The clinician also provided helpful feedback and insight into the significance of narratives told by participants.

## Results

All participants told stories illustrating canonicity and breach (Bruner, 1991) in relation to culturally available trans narratives. For all participants, they had come to understand their gender dysphoria as meaning they were TGD. They believed transition would alleviate their distress or improve their lives. When this did not happen, they questioned whether transition was right for them. Four narrative themes were developed to capture how participants made sense of this: the limits of medical transition; the long-term health implications; the social limits of transition; and detransition as an ongoing process.

### The Limits of Medical Transition

Transition was seen as limited in resolving dysphoria or distress. Three participants reported transition had intensified dysphoric feelings, the focus shifting to parts of their female anatomy they could not change. Luda (21, lesbian/asexual, autistic), who described herself as a “classic gender dysphoric child,” said:

I always, and still do ... have sexual dysphoria, so it felt like [testosterone] was helping that ... I liked it ... then I started thinking, “if I’m on hormones for 20 years, if I get top surgery, if I get bottom surgery, will I just find something else?” until they can take out all my bones and everything, I felt like I’ll just find something else that I’m not happy with ... it was just futile ... I’ll still be dysphoric

Her recalled childhood assessment that “transsexuality did not feel real enough for how I was feeling” seems to have played out in her adult experience. Luda’s narrative appeared to resist counter narratives (e.g., autism, lesbianism) to explain her experience, perhaps emphasizing the validity of her GD, or that “classic” cases may also not benefit from medical transition.

Susie (22, lesbian, autistic) described a similar experience with dysphoric feelings shifting and intensifying after starting testosterone, with the intense focus on her body perhaps heightening the disconnect between her embodied and desired self:

I’d stare in the mirror, and it was partly the dysphoria, and partly the dysmorphia, when I was outside in the real world, I was presenting myself as male, and then I would come back to my flat with the reality of my body as female, and that dissonance was really difficult to deal with ... I didn’t feel like a real person you know, and the dysphoria was just getting worse ... I was like, “I’m supposed to be feeling better.”

She felt isolated and unable to connect, not wanting to be seen as female. Her doubts resurfaced, “there was always part of me that was like the reason you think you’re male, is cos you read all those fan fictions, cos you got obsessed with these fictional characters, and that’s not real, that’s fantasy.” Struggling with her sexuality and feeling isolated, Susie had gone online, looking for reasons she did not fit in. However, as Susie’s mental health deteriorated, she saw her sex as immutable and realized transition was not the solution:

I was just thinking no matter what I do, I’ll always literally be female, like all the testosterone and the surgery it’s all kind of cosmetic and it’s altering how I appear but it’s not actually altering who I am, and it was who I was that I was unhappy with.

Anabelle (32, bisexual, autistic) said she had a “shifting self-hatred” for her “in between” body on testosterone and an “immediate hatred” for her mastectomy scars, resulting in regret regarding her choice of procedure. She contemplated the lengthy phalloplasty process and questioned whether it would achieve what she wanted:

I was also going through, that it would never be real ... it would never be a biological body, it would always be made ... I was just like “No, I don’t want to,” I wanted authenticity.

This connected with her deep desire for a relationship. She worried that her medically altered body might not feel “real” enough for a partner. While she reflected this might not have been accurate, it was a big issue for her, something she linked to autism-related “black and white thinking.”

Emma (29, bisexual), having had an “aesthetically freeing” double mastectomy, started to wonder why she was not feeling better:

I was passing 100% of the time ... I’ve achieved what I wanted to achieve ... and it felt great! ... but it was only a matter of time before I realized that testosterone and top surgery doesn’t magically get rid of the mental problems you have ... and I just remember sitting and thinking “I don’t understand why transition hasn’t worked the way everyone told me, why do I still feel so fucking horrible?” and then I went, “Holy shit, what if transition wasn’t what I needed”

Her earlier doubts about not having had childhood GD resurfaced, worrying she may have been caught up in the online transition “boom.” Attempting, unsuccessfully, to access therapy to discuss regret, she recalled saying “among other things, I’ve come to realize that I’ll never be a real man, I’m essentially living a lie.” For Sarah (27, lesbian, autistic), the seriousness of the surgery shocked her and became a turning point in her story:

I went off and had the mastectomy ... I had no idea what it was like to be seriously physically vulnerable ... this is ... bad enough, being here in this hospital bed, miserable as hell, having realized that this is horrific, imagine ... waking up with a phalloplasty that you didn’t want ... I was like, “This has to stop, because transition is not working.”

She felt betrayed by how transition had been portrayed within the trans community: “everyone tells you that mastectomy is basically fine, this is a big lie, a double mastectomy is a significant medical procedure.” Her recovery was slow and hard, and this left her questioning what she was doing, weighing the cost alongside the realization that the medical procedures could not change her body enough to satisfy her desire to be male:

One of my abiding feelings ... is that I still feel that I want to be a man, and unfortunately, I felt that when I was three years on testosterone and had a beard, and in the trans community you’re not allowed to want to be a man because you’re meant to believe that you are a man, but I didn’t, and I wasn’t.

In summary, participants seem to have detransitioned partly through a realization that the medical interventions had limits, to transform their bodies to male, to resolve their dysphoric feelings, or to overcome difficulties in their lives. Instead, they realized they could not change the reality of their sexed bodies, changes felt cosmetic, even harmful.

### The Long-Term Health Implications

This theme captures participants’ anxiety about the known and unknown effects of testosterone on their female anatomy, perhaps reflecting changing priorities as they matured. They highlighted their own lack of awareness about the impact of GAMSTs and they discussed ethical concerns for themselves, and for society. Susie shared her experience of hearing about the consequences of taking testosterone at her assessment:

Even then ... after six years (wanting to transition), I didn’t know that taking testosterone would mean your uterus would atrophy, and that you’d need a hysterectomy eventually, I had no idea, I didn’t really know what atrophy was.

Desperate to start, she shrugged this off, but the risk of infertility played on her mind. Charlotte (22, lesbian) had similar concerns, feeling burdened by the ongoing commitment to a medicalized pathway:

I remember having a conversation with my mum ... and I was like “Well, they told me about a hysterectomy so I could go through egg freezing beforehand,” and yeah, I remember then, just suddenly thinking, “Oh, this is just really tiring all of this.”

For Sarah, an increased awareness of her own family health risks as well as exposure to people living with long-term health conditions, highlighted the value of physical health and raised ethical concerns about the limits of medical knowledge:

I really worry about the ethics of people being on [testosterone] for years with no study of what it does to cardiovascular risk, to dementia risk ... we’ve created a group of people with very complex medical needs and I don’t want to have more complex medical needs than I have to have, it’s bad enough already.

Luda was concerned about dying young before she could make a difference in the world. She chose to prioritize this over her “delusion” of becoming male:

The medical unknowing was a big, big effect in why I wanted to stop it too, because I thought, “I want to do things with my life, I don’t want to die at a young age, having not done anything.”

In summary, participants were concerned about the medical implications of transition, the known, the unknown, and the long-term effects of medical interventions on their bodies and how this would affect their lives. The limits of current medical knowledge on the effects of testosterone on the female body weighed on their minds, creating anxiety.

### The Social Limits of Transition

This theme relates to the complex ways that transition changed participants’ social experience, affecting their sense of identity and political views. Most participants expressed having felt different to the “other girls” growing up, as Sarah said, “I wasn’t like the women around me, they all wore makeup and fancied boys.” They experienced themselves as different, compared to stereotypical ideals of womanhood and femininity, dominant within western cultures and their adolescent peer groups. However, once they identified as male, some felt different to “other men.” As Susie’s trans identity fell away, she evaluated the logic of herself as a man:

I was realizing I don’t actually relate to any men ... I was kind of terrified of them, and I was like, all I know about being a man is from TV, films, fiction, the men that I related to were fictional men, written by women for other women.

For others, the re-evaluation came once they were able to “pass” as male. Living “stealth” highlighted the ways in which Charlotte felt unable to connect with men or masculinity, and she disliked the masculinizing effects of testosterone. She felt uncomfortable in her body and awkward socially. It felt tiring and inauthentic trying to fit in as a man, suppressing her feminine mannerisms and interests, avoiding people “clocking me.” She said, “I suddenly realized that I couldn’t look at any of my childhood photos … I’m forever gonna be … hiding things from people.” As her horizons broadened beyond university, she questioned her future:

I started thinking “Oh, can I actually picture myself growing old as a man?” ... I was just kind of imagining all the women are friends, all their husbands are friends, and they just go down the pub and ... I just couldn’t relate to that at all.

She missed her femininity and close female relationships, feeling disconnected when female friends “othered” her:

They’d say something about men or whatever, and then they’d be like “Oh, but not you,” and I’d suddenly feel really alienated again ... cos in my head I’m subconsciously like, “I’m still part of this group” ... and I suddenly realized “Oh yeah, that’s me as well now.”

Similarly, post-mastectomy, Annabelle enjoyed the freedom of “passing” without binding. She felt more confident being treated as a “normal guy” and started university. As she started to connect with women for the first time, her sense of belonging changed:

I was starting to have my doubts ... I think it’s cos I was making friends with more girls, and now all my friends are girls ... and so I was starting to be accepted by all of them, and I was just like, “Well, what if I want to be more like them?”

She no longer related to men socially, finding it awkward to talk to them, “cos I didn’t feel like one of the guys, I felt like one of the girls.” This was reflected in how she felt comfortable expressing herself. She missed aspects of her femininity, and started admiring other women, saying, “I didn’t feel like that about the guys anymore … sort of seeing a guy and going, wow, I wish I looked like that, that had gone.” In fact, having a very feminine friend changed her view of what women could be:

I think it’s shown me that I’ve been a little bit prejudiced I guess, against certain looks of people ... you can have all these nice makeups and nails, and she’s not an airhead, she’s very clever, she’s really hard-working.

This suggests that escaping narrow stereotypical versions of womanhood might have been a factor in Anabelle’s transition. Connecting with women as an adult led to a re-evaluation of previous assumptions. Sarah, who had developed a trans identity through blogging, activism, and in her dating life, described a “crunch point” once she “passed” as a man:

I was like, “Do I want to be a man in the real world?” being a man in a generic office is very different to being a man in your hyper-liberal student bubble … people won’t understand that I’m trans … I was like “Christ, people just think I’m a man, this is very depressing and not what I signed up for.”

She felt “deeply miserable about women not recognizing me as a woman,” not wanting them to treat her as a man. Yet she did not feel like a “normal woman,” identifying most with trans men. She struggled to live up to male social norms, meaning she was read as a gender nonconforming or gay man. Neither social role felt a good fit, contributing to her disillusionment:

I don’t want to have to enter the dating ... marketplace as a man because I don’t know how to be a straight man... I didn’t feel equipped to date women as a man even though this was my deep desire in life ... I’m realizing ... I couldn’t be a woman, but I can’t be a man either, but I don’t want to become genderqueer ... I don’t think that solves anything for me … and I stopped injecting testosterone.

Given the social nature of gender roles, Sarah raised the potential that autism may have made it more difficult for her to adapt her behavior to fit within cisnormative societal expectations:

Assuming I had transitioned and wasn’t autistic I may well have made a better stab of being a man, because I would have been more able to have social graces like men, I know trans men who are in a great spot ... they hit off perfectly like nice young man.

After stopping, Sarah found herself caught between an external reality of living as a man, and a home reality as a carer. This added to her frustration with transition:

It was like, “I have put so much effort into becoming a man ... and it’s done nothing ... you can change how you look, you can change how you sound, you can change your body, you really can do all those things, you cannot undo a lifetime of socialization, and you cannot utterly transform where you are and who you are as a person,” and the icing on the cake was being told that I was then a man and enjoying male privilege.

She gave many examples of the way in which gendered ideas about social roles and behaviors created new challenges to those she had experienced as a gender nonconforming lesbian. For example, navigating male–female typical sexism as a trans man, or being told that as a female-attracted person, she should have sex with trans women. The cumulative effect meant she started to see trans narratives (i.e., that trans men are men) as “hollow” and restrictive:

Detransition has for me meant leaving behind the ideology that led me to tell myself a lot of things that I did not believe .... what I resent is being told that you’re not also biologically male or biologically female ... it’s this re-writing of history ... it stops you identifying yourself as a complex person if someone says “Oh every bit of gender nonconformity that I have is totally explained by transition I am just a totally normal man now” I found that very repressive.

Sarah and Luda also struggled to make sense of their identity as trans alongside their identities as feminists. Luda said:

If you’ve got people who have experienced FGM (female genital mutilation) and you’re having to have a pronoun circle ... what’s that saying to them? that sex-based discrimination doesn’t exist? ... and I was going to a group for trans people, and because I was different, I was happy at this point being female, supporting sex-based rights, a lot of trans people were saying, “you’re wrong for doing this, you’re disgusting.”

She felt frustrated with the trans community, increasingly aligning with feminism, perhaps wishing to avoid being seen as misogynistic, reflecting feminist narratives in the wider culture:

I was thinking, “although I have a masculinized body, I am a woman, I shouldn’t be ashamed of that,” I feel like a lot of people who really, really strongly are against being female even after years of transitioning are ashamed to be women or something, and I thought “No I am this, I should be happy with this.”

In summary, participants found their social experience shifted in transition. Stepping into a male role, some felt increasingly identified with women, either socially or politically. Autism seemed to play a part for some in how they were interpreting their experience, perhaps reflecting the concrete and binary thinking common in this group. Awareness of their sexed bodies may have heightened these feelings, and some felt uncomfortable with trans narratives that prioritized gender identity and minimized the importance of sex and socialization.

### Detransition as an Ongoing Process

This theme articulates the sense that detransition was a complex, individual, and ongoing process. All participants expressed that it took time to come to the decision, doubting privately, before acting. Some faced feelings of shame or embarrassment regarding their decisions, or guilt that they had involved loved ones financially or emotionally. Emma said:

Having to admit so publicly that I made such an enormous mistake was just (pause) I was like “I’ll just I’ll live as a trans man it’s fine” ... but there’s only so long you can keep pretending.

Once she told loved ones, she took time to reflect, “transition regret” making it hard to trust herself. Eventually, she withdrew from education and employment to avoid a second public transition. Annabelle described a similar process, wanting to be sure of herself:

I basically ignored it, because I was like ... this is done, I’m on the (phalloplasty) waiting list, that’s how it is, it went over and over in my head .... probably the best part of a year ... and I just went, “it’s not too late, I don’t have to do that, I can get my old life back, if that’s what I want.”

Others, feeling stuck, shared how important it had been to hear about detransition. Sarah heard about a friend who had shared her theory of trans-ness, someone with similar experiences of identity instability:

It did make it feel like a real thing that I could do ... cos I’d been toying with the idea for years ... but I hadn’t known that anyone actually detransitioned ... I didn’t know you could actually do it and ever pass as a woman again ... to see someone else do it, I was like “God, I do have the option if I want it.”

Susie, her mental health spiraling, accessed therapy for an eating disorder, and started making connections between the feelings that were making her starve herself and the push for transition. She watched her friend’s detransition videos out of “morbid curiosity.” The stories resonated and she reached a turning point:

It just got to the point where it was like, “I might be ... changing my body needlessly ... cos I’ll never get to a place where I can be satisfied that I’m male enough ... and there’s a possibility that in the future I might regret this ... and if there’s a possibility that I am wrong, then I can’t carry on” ... it was terrifying realizing that, it felt like the floors had fallen away from me, that identity that I’d built for myself just wasn’t holding up anymore, it was really scary, but I knew it was what I had to do, so I stopped testosterone.

Since then, Susie has spent time trying to understand herself in therapy, missing the certainty of a “cure.” Overall, she feels better, that she can be a part of society now she is not trying to be someone she is not. She connected with other detransitioned women online, which gave her a sense of hope for the future and helped her to cope when she felt overwhelmed by regret, loss, and thoughts that she had ruined her life. She has enjoyed re-finding herself as a lesbian now she no longer identifies as TGD, her sexuality confusion falling away with this identity. For her, it was important to hear other butch lesbians talking about their experiences of GD as this helped her to realize, “I didn’t have any inherent gender identity, it was just the way I was interpreting my feelings.” This new framing for her experience of GD, lacking prior to her decision to transition, has been helpful in allowing her to move forward, although she misses the trans community:

Cos now when I’m feeling like I want to retransition, a lot of it is feeling like I’ve not really got anyone to relate too anymore, cos a lot of the people that feel the same way as me, they identify as trans so ... I’ve lost that community I had ... the feeling of connection, now I’m a little bit back with that feeling isolated, like I don’t fit in.

Charlotte, not realizing she could “go back,” tried to “make the best of it, to be the best guy I could.” Finding a detransition subreddit provided her with an alternative, yet the decision felt much harder this time, because of the guilt she felt, because “you’ve gotta admit you were wrong … you then realize what you’ve lost, what you’ve done to yourself.” She was worried about, “the idea of having to face another kind of in-between period, cos that was the horrific period before.” Finding out there were things she could do made the decision clear, although she has struggled with regrets, she said, “it kind of was worth it to get to where I am now, I’d say I’m probably happier than I’ve ever been.” Two years on she said, “the thing that does bother me the most is my chest … cos I’m missing a part of myself now.” Reconstruction options did not feel viable. This was affecting intimacy with her partner, and she reflected she may have enjoyed having her chest now, that perhaps she needed more time to “grow into” her body.

Practical information about what would happen and what could be reversed was key for several participants. However, Anabelle reflected that reversing changes is “all time and money.” She was mourning the loss of her singing voice, yet she said, “it’s different this time, I feel more empowered … I used to feel it was socially difficult to be female, but now I’m feeling really sort of, I can be that strong, empowered, beautiful woman.” In contrast, while Emma valued practical advice from detransitioners, she found feminist reassurances she was still a “strong, beautiful woman” misunderstood the heart of her distress. She felt a lot of anger and resentment toward the gender clinic, meaning she did not return for support. Yet therapists she approached were not willing to talk about detransition, even viewing her as transphobic, leaving her isolated. Social and family support was therefore invaluable to her, as to other participants. She described ongoing periods of feeling very low, yet said she was growing more comfortable with gender nonconformity and the social confusion that could result:

After everything that’s happened, I still don’t understand why I am uncomfortable being a woman ... but it’s become easier I think ... I regret having gone through transition, but I’m growing more ... comfortable with a more male appearance.

Similarly, Luda said, “I just don’t speak about gender or anything … I think most of the people on my course probably think I’m male and some people think I’m female, I don’t question it.” Her quest was to manage GD and to live a happy, meaningful life, no longer thinking about gender. She had explored a life of religious devotion pre-transition, saying:

Now I’m pretty much an asexual person so I thought there’s no need to really worry about my body in that respect, if I can control it myself ... I just want to live my life, do what I can, and just not think about my body.

Sarah was also accepting her masculinized appearance, having previously felt trapped in heteronormative ideals of masculinity or femininity. She had also found other ways to connect with her body which eased dysphoria:

I used to get quite miserable about the fact that you need to look like either a man or a woman ... now I’ve been growing my beard out ... I don’t think it makes me any less female, I just look like this ... and I’m a lot happier with my body ... running is so good because all I’m bothered about is will my body run ... it’s just so good for you to move and to do a sport.

Most participants experienced ongoing GD, some about the changes made during transition (reverse dysphoria). Sarah described intermittently feeling the “body horror” because part of her is missing, and at other times, “the siren call of transition,” imagining she would feel better back on testosterone. Overall, she reflected she now prefers to find other solutions for her distress:

Stopping looking in gender for the solution and trying to sort myself out more generally is the only solution for me, but it’s not fun compared to transitioning ... gender is the place my problems manifest ... it is not the place they come from, how do I actually solve them so that I can have a happy and healthy fifty years rather than a chaotic 10 minutes?

In summary, detransition was a complex and personal process, often involving strong emotions of sadness, grief, and guilt, for what transition had cost them or their families. Some felt anger, or resentment toward the gender clinic, regretting their decision to transition or particular GAMST outcomes. Others seemed more accepting that transition had been part of their process, particularly when they felt able to move forwards now. Participants described challenges navigating life with a masculinized appearance in a world that expects people to fit into a binary category. They struggled with an altered relationship to their body, some struggled with reverse dysphoria or ongoing GD related to their birth sex/gender, lamenting the costs and time involved in reversing changes, or the lack of acceptable or any options for this. Some found that the difficulties they hoped transition would resolve were still there. However, most felt that working on themselves and accepting themselves would lead them to greater happiness and personal freedom, including feeling free from stereotypical gender or sex-based norms.

## Discussion

This study used thematic narrative analysis to explore the stories of people who detransitioned following a medical or surgical gender transition in the UK. Four narrative themes were developed to capture how they made sense of their detransition.

The limits of medical transition reflected the participants’ experience that changing the body had limits for resolving dysphoria. While there is ongoing debate regarding the appropriateness of diagnostic criteria for gender incongruence (Beek et al., 2016), participants in this study had understood their distress as indicating they were the opposite sex/gender (“trapped in the wrong body”) and believed they needed to medically transition to resolve this. This finding has been echoed in other studies, e.g., MacKinnon et al. (2023b) also found that participants thought that pursuing a medical transition would improve their mental health, conforming with dominant transnormative ideology, which one participant in this study referred to as being caught up in the transition “boom.” However, as with participants in other studies (e.g., Sanders et al., 2023; Vandenbussche, 2022) and case studies (Herzog, 2017; Lev, 2019; Marchiano, 2020), after what has been termed a “honeymoon period” (Jorgensen, 2023), participants’ mental health once again declined. This confirms that GD does not always overlap with, or imply, a TGD identity (Zucker, 2019) and that GAMSTs may not resolve GD. Indeed, after detransitioning, participants in this study described finding alternative solutions to ease gender-related distress, rather than medical interventions. This ability to find alternative ways to cope with gender-related distress was one of two (the other being political/ideology concerns, also relevant for participants in this study) most commonly cited reasons cited in two online surveys with detransitioners conducted by detransitioners (Hailey, 2017; Stella, 2016; cited in Hilderbrand-Chupp, 2020).

In the theme detransition as an ongoing process, some participants also realized that the distressing feelings they had been experiencing were not GD but down to other issues, a finding echoed in other studies where participants re-evaluated the cause of their distress, e.g., because of past trauma (Gould et al., 2023), internalized homophobia (Littman, 2021; Vandenbussche, 2022), depression or internal misogyny (Pullen Sansfaçon et al., 2023; Vandenbussche, 2022). Participants engaged in a process of meaning making and seeking alternative narratives to GD, and alternative ways to manage it, that allowed them to feel more comfortable, as noted in other studies (Pullen-Sansfaçon et al., 2023; Sanders et al., 2023). For example, GD might be a part of lesbian, gay, or bisexual identity development (Patterson, 2018) as people reject heteronormative gender stereotypes. People with GD may not wish to medicalize due to health concerns, regardless of TGD identity.

Participants in this and other studies (Jorgenson, 2023) described a lack of information about the consequences of transition, with participants in this study concerned about the medical side effects of interventions (e.g., vaginal atrophy, infertility) and limits in current medical knowledge (e.g., the long-term effects of testosterone on the female body, such as possible cardiovascular risk or dementia). This links to a distrust of gender care specialists, feeling betrayed by them, with participants in this and other studies not returning to clinics for further support (Sanders et al., 2023). Health concerns have frequently been found to contribute to detransition decisions (Exposito-Campos et al., 2023; Littman, 2021; Vandenbussche, 2022) and participants may feel a heightened vulnerability as health is an area where biological sex matters (Shapiro et al., 2021). Most health research has focused on male-bodied people (Arnegard et al., 2020), which may not correspond with the needs of male-identified female-bodied people, and Littman (2021) found that health implications were of greater concern to detransitioned women than detransitioned men.

Disappointment or dissatisfaction with the results of GAMSTs has been linked to detransition and regret (Exposito-Campos, 2023), including in people who continue to identify as TGD (Bustos et al., 2021; Cain & Velasco, 2021; Graham, 2017). Regret in this context can be related to the aesthetics, functionality, or medical complications arising from the procedures (Bustos et al., 2021; Pfäfflin, 2019). Such regret was mentioned by participants in this study in relation to dissatisfaction with mastectomy scars or feeling vulnerable and scared after mastectomy, or concerned about how body changes might impact dating. Over a third of female detransitioners have been reported to have had a double mastectomy and this number is believed to be increasing (Gribble et al., 2023), but alongside this, there is a lack of support or guidance (e.g., with regard to breastfeeding) provided for detransitioned women who have undergone this procedure (Gribble et al., 2023). One participant in this study spoke about feeling lied to by the trans community and medical professionals as they felt the significance of this procedure was downplayed, contributing to a move away from identifying as TGD.

Some participants articulated that they disliked masculinizing changes (e.g., hair growth) or felt reserve dysphoria (Exposito-Campos et al., 2023; Pullen Sansfaçon et al., 2024) about bodily changes that happened following medical intervention. Littman (2021) similarly found that just over half of the females in her study, compared to just over a quarter of the males, reported that the changes following medical intervention felt “too much.” However, some participants spoke about how detransition meant they no longer felt trapped, needing to look either masculine or feminine, or be viewed by others as only one or the other. Similarly, the youth in Pullen Sansfaçon et al.’s (2024) study experienced a sense of liberation when first detransitioned, precisely because of the freedom it gave them to disengage with expected transnormative binary gender norms. Participants in Pullen Sansfaçon et al.’s (2023) study reported that detransition was not experienced as a shift in gender, but as a change in the meaning of gender. In Pullen Sansfaçon et al.’s (2024) study, participants also said they no longer felt like they belonged to any gender categories after detransition, which was the case for one participant in this study, while others felt that socially their gender/appearance mattered less and they identified more with their biological sex. Hilderbrand-Chupp (2020) describes how detransitioned people live in the gap between the concepts of “trans” and “cis” gender. It is important for clinicians to explore how people experience their bodies and identities throughout the detransition process. This may change throughout the transition and detransition experience, at different time points, and in relation to GAMSTs, and this might impact well-being and support needs.

Participants in this study came to view medical/surgical interventions as cosmetic, along with a realization that they could not become biologically male or male bodied, something they desired and were told they were “meant to believe” in transgender communities. In Littman’s (2021) study, over a third detransitioned female participants felt dissatisfied with physical changes that were “not enough.” Sanders et al. (2023) also reported that people who had detransitioned expressed a sense of feeling inauthentic in their new gender, connected to a realization that they would never be biologically the opposite sex. Some participants in this study found that stepping into the social role of a man did not fit for them, that they felt inauthentic. This shift demonstrates a change in beliefs about gender, sex, and transition (Expositio-Campos et al., 2023), and Littman (2021) reported that 60% of her participants detransitioned because they became comfortable identifying with their natal sex following a shift in their construction of what this meant. MacKinnon et al.’s (2023b) participants viewed detransitioning as allowing them to feel “authentic” again, offering a new opportunity for gender self-exploration, expression, and expansiveness. A participant of this study described that while experiencing regret, she also felt “happier than I’ve ever been” having gone through transition and detransition. Pullen Sansfaçon et al.’s (2023) participants also described experiencing both regret and a sense of relief and happiness, greater freedom, and alignment with the self. Some participants in this study described how a narrow stereotypical view of womanhood might have influenced their decision to transition, but that detransitioning had widened this view, e.g., accepting they might be seen as either male or female. Indeed, Sanders et al. (2023) found after detransitioning, participants compared their physical appearance against both masculine and feminine cisgendered norms. Katz-Wise et al. (2023), Cohen et al. (2022), and MacKinnon et al. (2023b) all report regular gender identity shifts while people detransition, with a significant proportion of people adopting a nonbinary identity (which might not become a fixed “final” identity).

However, the shifting meaning of gender also meant that some participants experienced a lack of belonging in any group, feeling isolated because of not wanting to be misgendered while trying to live as a man, privately struggling with their sexuality within these changes, unable to connect with other men or masculinity, and/or no longer belonging to female groups. In addition, a participant described feeling isolated during detransition, as she withdrew from employment and education to avoid a second public transition. Another participant described feeling isolated after detransition and missing the solace of that transgender community, and for one participant, trying to find a therapist to discuss detransition compounded this sense of isolation as she felt viewed as transphobic. The sense of isolation that detransitioning brought that was clearly expressed by multiple participants in this study has not come across as strongly in other studies, although a sense of social exclusion has been discussed (MacKinnon et al., 2022b; Pullen Sansfaçon et al., 2023; Vandenbussche, 2022). A sense of community was sometimes refound for participants through connecting to online detransition content, giving them a new understanding of what was possible and a sense of hope. MacKinnon et al. (2023b) found that this was particularly so for detransitioned women, who resonated with stories, where detransitioned men did not connect with them in the same way. Online detransition communities have been highlighted as providing essential support in other studies (Littman, 2021; Littman et al., 2024; MacKinnon et al., 2023b), particularly when support is lacking from medical professionals or not wanted from professionals because of mistrust.

With regard to the social limits of transition, it is important to note that for the female participants in this study, they did not refer to discrimination from others as a reason for detransition, although one reported it was a reason they would not retransition. Discrimination has been reported as a social reason for detransition in Exposito-Campos et al.’s (2023) review of the literature. This was also found for MacKinnon et al. (2023b), who found that discrimination was a detransition motivating factor exclusively for trans women and many of these participants later retransitioned when the discrimination stopped. Discrimination as a trigger for detransition might therefore not apply as much for females, which might be because transfeminine people have been found to be more vulnerable to discrimination and transmisogyny (Bradford et al., 2013; Fuller & Riggs, 2018).

In line with previous studies (Littman, 2021; Pullen-Sansfaçon et al., 2023; Turban et al., 2021; Vandenbussche, 2022), this study illustrated the diversity, complexity, and individual nature of detransition experiences, captured in the theme detransition as an ongoing process. Echoing the importance of holding a coherent worldview, found by Littman et al. (2024) and Exposito-Campus et al. (2023), some participants expressed a tension between trans ideology and feminist values, problematizing theories of transgenderism that prioritize gender self-identification over birth sex. This reflects ongoing debate at a sociocultural level regarding issues such as trans people’s access to single sex spaces where there is concern that this detrimentally affects females (Stock, 2021). When living as men, participants in this study also found they had to navigate sexism expressed by other men, or avoid being seen as misogynistic because of holding a male identity—positions that clashed with their feminist values. Political views have been found to be an important factor in detransition (Exposito-Campos, 2021; Herzog, 2017; Kermode, 2019; Stella, 2016; Turban & Keuroghlian, 2018; Vandenbussche, 2022), and a sense of values incongruence could be a factor that leads some people to consider alternatives to GAMSTs for managing GD.

Some participants named autism as interacting with their experience and their narratives often suggested concrete and binary thinking common among autistic people, reflecting the heteronormative and dominant binary societal view of gender, and the related stereotypical ideals of femininity and masculinity. Autism may have heightened their sensitivity to their experiences of difference (e.g., to being “in-between;” to perceptions regarding social group belonging) especially in relation to the limits of medical transition and the social limits of transition themes. Researchers have suggested that some autistic people may struggle to read social cues regarding social roles and gender norms, or to be flexible in interpreting these (van der Miesen, et al., 2016, 2018); however, Cooper et al. (2023) found that this was only a view held by clinicians, and that autistic adults and young people stated autism did not impact upon their understanding of gender. However, autistic people, carers, and clinicians agreed that the features of autism compound gender dysphoria. There have been no studies to date specifically focused on how detransition and autism intersect, although it has been picked up as important in this study and others (Hall et al., 2021; Littman, 2021; MacKinnon et al., 2022a; Vandenbusshe, 2022).

### Clinical Implications

The option to detransition has grown in people’s awareness, with an explosion of information on social media from detransitioners in recent years (MacKinnon et al., 2023b). This study and others have found that detransitioners often turn to social media for support and information when this is found lacking in social networks and care providers. It is therefore essential that they can access appropriate information and support regarding the physical, practical, psychological, and social aspects of detransitioning (Vandenbussche, 2022). MacKinnon et al. (2023b) suggest it would be useful to provide peer-led discussion groups and resources to support people to navigate key topics pertinent to detransitioning, such as finding knowledgeable care providers, disclosing detransition to others, physical and sexual health, etc. Such groups and resources help people feel less isolated, as was found to be the case in this study.

While detransition is not synonymous with regret (Exposito-Campos, 2021), it is important to acknowledge that some people may have very difficult and painful feelings about their transition and may struggle with irreversible physical changes (“reverse dysphoria”). However, participants in this study, and others, reported that transition and detransition could be part of an important process in the search for authenticity and happiness. It is important that clinicians do not assume which emotions may accompany a detransition and are open to exploring clients’ potentially conflicting emotions, understanding that these might change over time.

A key finding of this and other studies is that GD might not be synonymous with TGD identity but might have other causes. This highlights the importance of holistic, developmentally informed assessment and formulation of factors that might be contributing to sex or gender distress (Churcher Clarke et al., 2019). Other studies have found a thorough assessment was reported as lacking (e.g., MacKinnon et al., 2023c).

These findings point to the importance of exploring models of sex and gender used by those who detransition (or transition) (Levitt & Ippolito, 2014), particularly as the terms sex and gender are often conflated (Vegter, 2013). Indeed, the language around gender-affirming care, born out of a desire to validate TGD identities, can obscure or minimize the nature of medical interventions (Stock, 2021). It is essential to use clear language to help people to gain a realistic understanding of the consequences of starting or stopping medical interventions, and more knowledge is needed about what these consequences might be.

### Strengths and Limitations

The strengths of this study are the use of a precise definition of transition and detransition, inclusive recruitment practices, rigorous and transparent procedures, and collaboration with an expert by experience increasing trustworthiness and credibility. However, personal narratives were captured at one time point and experiences may have been reinterpreted or omitted as part of an evolving process of narrative identity development (McAdams & McLean, 2013). The wider context of their experience, the anticipated audience, and the relationship between interviewer and interviewee will have influenced the story told by participants and this write up and will likely have been influenced by the researchers’ identities as cisgender women (e.g., Galupo, 2017).

Given the findings and participant demographics, this study may have reached a similar sample to Littman (2021) and Vandenbussche (2022). Reaching different populations is key to understanding diverse experiences and needs, which should be addressed in future research. Participants in this study were all White and born in the UK, all were registered female at birth, and identified as lesbian and bisexual, with four diagnosed with autism. All had at least started university and were aged 21–32 years old when they took part in the study. All had started GAMSTs in their late teens, or twenties. While the qualitative design and small, restricted sample means findings are not generalizable, the results may be transferable to similarly situated individuals (Lincoln & Guba, 1985). This study did not explicitly collect data on year of transition or detransition, and given the rapidly changing cultural context, this may have been relevant. The results of this study may had differed had there been a larger and more diverse sample, for example, including detransitioned men or participants who had detransitioned but later retransitioned. We did not explore shifts in gender identity or explore the shifting meaning of gender in any depth, but this appears to be an important theme emerging from recent literature as a challenge to transnormative binary ideas of gender (e.g., Gould et al., 2023; Katz-Wise et al., 2023; MacKinnon et al., 2023b; Pullen Sansfaçon et al., 2023, 2024). Future research could focus on the freeing of gender expectations and norms that detransitioners are describing, for the benefit for those constrained by transnormative or cisgendered binary gendered expectations.

### Conclusion

This research adds an in-depth analysis to the previous literature on detransition. Participants detransitioned because they found medical transition had limits to resolve distress, or to change their sex. The ongoing health implications and associated risks were also a factor for most. Participant perception of social group belonging often shifted after transition, sometimes linked to feminist values, or perhaps reflecting the normal developmental experiences and trajectory of adolescence and early adulthood as young people explore their identities in different social contexts. It is also possible that the experience of the physical limits of transition to transform them, left the social aspects feeling hollow, increasing social dysphoria related to the new gender. Detransition was a complex and individual, ongoing process, with a range of needs expressed. Implications for clinical practice with people considering transition and detransition were discussed.

## Data Availability

The interview transcripts are not available to preserve anonymity.
